# Serum 25-Hydroxyvitamin D and Insulin Resistance in Apparently Healthy Adolescents

**DOI:** 10.1371/journal.pone.0103108

**Published:** 2014-07-29

**Authors:** Dong Phil Choi, Sun Min Oh, Ju-Mi Lee, Hye Min Cho, Won Joon Lee, Bo-Mi Song, Yumie Rhee, Hyeon Chang Kim

**Affiliations:** 1 Department of Public Health, Graduate School of Yonsei University, Seoul, Korea; 2 Department of Preventive Medicine, Yonsei University College of Medicine, Seoul, Korea; 3 Department of Internal Medicine, Yonsei University College of Medicine, Seoul, Korea; 4 Cardiovascular and Metabolic Diseases Etiology Research Center, Yonsei University College of Medicine, Seoul, Korea; Children's National Medical Center, Washington, United States of America

## Abstract

**Purpose:**

Vitamin D deficiency is a common condition that is associated with diabetes and insulin resistance. However, the association between vitamin D and insulin resistance has not been fully studied, especially in the general adolescent population. Therefore, we assessed the association between serum 25-hydroxyvitamin D [25(OH)D] level and insulin resistance among apparently healthy Korean adolescents.

**Methods:**

A total of 260 (135 male and 125 female) adolescents in a rural high school were assessed for serum 25(OH)D, fasting plasma glucose, and insulin. All of the participants were aged 15 to 16 years old, and without known hypertension or diabetes. Serum 25(OH)D was analyzed both as a continuous and categorical variable in association with insulin resistance. Insulin resistance was estimated by homeostasis model assessment (HOMA-IR). Increased insulin resistance was operationally defined as a HOMA-IR value higher than the sex-specific 75th percentile.

**Results:**

In male adolescents, every 10 ng/ml decrease in 25(OH)D level was associated with a 0.25 unit increase in HOMA-IR (p = 0.003) after adjusting for age and BMI. Compared to those in the highest quartile, male adolescents in the lowest 25(OH)D quartile were at significantly higher risk for insulin resistance: unadjusted odds ratio 4.06 (95% CI, 1.26 to 13.07); age and BMI adjusted odds ratio 3.59 (95% CI, 1.03 to 12.57). However, 25(OH)D level, either in continuous or categorical measure, was not significantly associated with insulin resistance among female adolescents.

**Conclusions:**

This study suggests that serum 25(OH)D level may be inversely associated with insulin resistance in healthy male adolescents.

## Introduction

Vitamin D is an important fat-soluble vitamin that functions in calcium and phosphorus homeostasis and affects bone mineralization. Functions of vitamin D are not limited to skeletal effects, and a non-skeletal action under active investigation is the role of vitamin D in insulin or glucose metabolism [1]. Vitamin D status may play a functional role in glucose homeostasis as *in vitro* and *in vivo* studies have provided biological evidence of its effects on insulin secretion and insulin sensitivity [2]–[6]. Several epidemiological studies have reported associations between low vitamin D status, as indicated by circulating serum 25-hydroxyvitamin D [25(OH)D], and the risk of type 2 diabetes [7]–[10], although others have not found such an association [11], [12]. A study of the Third National Health and Nutrition Examination Survey (NHANES III) showed an inverse association between serum 25(OH)D level, insulin resistance, and diabetes in non-Hispanic whites and Mexican Americans, but not in non-Hispanic blacks [13]. Serum 25(OH)D level was inversely associated with insulin resistance in a study of Chinese individuals [14], but was not associated with insulin resistance or beta cell function in a study of the Canadian Cree [12].

An association between hypovitaminosis D and insulin resistance has also been reported in overweight or obese adolescents [15]–[19], but the association was not observed in the general adolescent population [20]. The association between vitamin D and insulin resistance has not been fully explored, especially in healthy adolescents. Therefore, we investigated the association between serum 25(OH)D level and insulin resistance among apparently healthy Korean adolescents.

## Materials and Methods

### Study population

We conducted a cross-sectional analysis of baseline data collected for an adolescent cohort study. All first-year students of a high school in a rural community in South Korea were invited to participate in the cohort study. Among the total 283 first-year students, 268 students agreed to participate in the study between May 30 and June 22, 2011. After exclusion of seven individuals who were missing serum 25(OH)D measurements and one individual with diagnosed hypertension, 260 students were included for this analysis. All participants were aged between 15 and 16 years (mean age 15.9 years) and had not been previously diagnosed with diabetes mellitus. Written informed consent was obtained from each participant and his/her parent or guardian. Informed consent forms were distributed to eligible students at least one week prior to the examination, so the participating students and their parents had enough time to understand the purpose and process of the study. On the day of examination, research staff checked whether each consent form was completed and signed by the student as well as his/her parent or guardian. The study protocol and consent procedure was approved by the Institutional Review Board of Severance Hospital at Yonsei University College of Medicine (Approval No. 4-20100169).

### Measurements

Health-related lifestyle, personal and family medical history were evaluated with self-report questionnaires. Smokers were defined as participants who smoked more than 100 cigarettes in their lifetime. Drinkers were defined as participants who consume alcoholic beverage at least once a month over the last year. Regular exercise was defined as physical activity on a regular basis (at least once a week for 30 minutes at moderate intensity) regardless of indoor or outdoor exercise. Anthropometric measures, performed using the same devices throughout this study, were taken by a trained examiner. Standing height was measured to the nearest 0.1 cm on a stadiometer, and body weight was measured to the nearest 0.1 kg on a digital scale (Seca 763; SECA, Hamburg, Germany) while wearing the school uniform. Body mass index (BMI) was calculated as weight in kg divided by squared height in meters (kg/m^2^). Waist circumference (WC) was measured to the nearest 0.1 cm at the level of the superior iliac crest at the end of a normal expiration. Resting blood pressure and pulse rate were measured with an oscillometric device (Dinamap 1846 SX/P; USA). Participants were seated in the examination room for at least five minutes before blood pressure measurement, and then an appropriately sized cuff was applied snugly around the right upper arm at heart level. Cuff size was chosen for each subject according to mid-arm circumference. Two readings at five minute intervals were obtained and averaged to determine systolic blood pressure (SBP) and diastolic blood pressure (DBP) for each individual. When the two readings differed by ≥10 mmHg, additional readings were obtained after five minutes, and the last two readings were averaged.

An overnight fasting blood sample was collected after at least an 8-hour fast. Serum concentrations of total cholesterol, triglycerides, high-density lipoprotein (HDL) cholesterol, aspartate aminotransferase (AST), and alanine aminotransferase (ALT) were measured by enzymatic methods with an autoanalyzer (7600 Autoanalyzer, Hitachi, Tokyo, Japan). Serum 25(OH)D was measured by radioimmunoassay (BioSource, Nivelles, Belgium) with inter-/intra-assay coefficient of variations (CVs) of 5.2% and 3.3%, respectively, and a reference range of 7.6 to 75.0 ng/ml. Fasting plasma glucose (FPG) level was measured by the glucose hexokinase method. Fasting plasma insulin level was measured by the radio immunometric method. Insulin resistance was assessed by homeostatic model assessment (HOMA-IR), calculated as the product of the fasting insulin level (uIU/mL) and the fasting glucose level (mg/dL), divided by 405.

### Statistical analyses

All statistical analyses were performed separately for males and females, because there were significant sex-differences in serum 25(OH)D levels and a significant interaction (p = 0.019) between sex and 25(OH)D on HOMA-IR levels. Differences between groups in quantitative variables were evaluated by student's *t*-test, *x* ^2^-test, or Wilcoxon rank sum test. Spearman correlation and linear regression analyses were used to examine the relationship between variables. Both categorical (quartile groups) and continuous measures of 25(OH)D were analyzed in association with fasting glucose, insulin, and HOMA-IR levels, with and without adjustment for age and sex. Multiple logistic regression models were also used to evaluate the odds ratio and 95% confidence interval for increased insulin resistance (defined as HOMA-IR ≥75 percentile) for quartile groups of serum 25(OH)D as well as for 10 ng/ml decrease of 25(OH)D. In order to assess the independent association between serum 25(OH)D, two adjusted models were employed; first for age and BMI, then for cigarette smoking, alcohol consumption, and regular exercise. All analyses were 2-tailed and performed using SAS system version 9.2 (SAS Institute, Cary, NC, USA).

## Results

The characteristics of the study participants are given by sex and in total. Male participants had significantly higher BMI, SBP, AST, ALT, FPG, and 25(OH)D, but significantly lower HDL cholesterol level than female participants. None of the adolescents had fasting glucose higher than 110 mg/dl. Thirteen adolescents (5.0%) were current smokers, 16 (6.1%) consumed alcoholic drinks at least once a month, and 219 (83.9%) exercised on a regular basis (Table 1). Although the participants were recruited from a single high school, their anthropometric distributions and serum 25(OH)D levels were similar to those in the 2007 Korean National Growth Charts and the 2009 Korean National Health and Nutrition Examination Survey. Mean height was 172.8/160.7 cm (male/female) in this study, and the corresponding value from the growth charts was 173.3/160.7 cm. Mean body weight was 66.4/55.5 kg (male/female) in this study and 65.8/54.1 kg in the growth charts. Mean serum 25(OH)D level was 17.0/14.4 ng/ml (male/female) in this study and 17.2/16.4 ng/ml in the Korean National Health and Nutrition Examination Survey [21], [22].

**Table 1:** Participants characteristics.

Variable	Total (n = 260)	Male (n = 135)	Female (n = 125)	*p-value*
Mean ± SD				
Age, year	15.9±0.3	15.9±0.3	15.9±0.3	0.274
Body mass index, kg/m^2^	21.7±2.9	22.2±3.2	21.2±2.5	0.011
Systolic blood pressure, mmHg	103.4±11.5	109.4±10.8	96.8±8.4	<.001
Diastolic blood pressure, mmHg	57.9±5.9	58.4±6.1	57.4±5.8	0.204
Total cholesterol, mg/dl	156.9±27.2	150.0±26.6	164.5±26.0	<.001
HDL cholesterol, mg/dl	45.6±8.8	43.1±7.7	48.3±9.1	<.001
Triglycerides, mg/dl	83.1±30.3	85.6±30.7	80.4±29.8	0.066
Aspartate aminotransferase, IU/l	20.4±5.7	21.9±7.1	18.7±2.9	<.001
Alanine aminotransferase, IU/l	15.9±9.2	18.5±11.8	13.0±3.2	<.001
Fasting glucose, mg/dl	84.5±6.2	86.0±6.3	82.9±5.8	<.001
Fasting insulin, uIU/mL	8.54±3.12	8.67±3.44	8.42±2.76	0.995
HOMA-IR, mg/dl/mL/uIU	1.79±0.71	1.86±0.79	1.73±0.61	0.314
25(OH)D, ng/ml	15.8±7.2	17.0±6.9	14.4±7.3	<.001
Number (%)				
Smoking (≥100 cigarettes)	13 (5.0)	12 (8.8)	1 (0.8)	0.546
Drinking (≥1 time/month)	16 (6.1)	10 (7.4)	6 (4.8)	0.160
Regular exercise (≥1 time/week)	219 (83.9)	120 (88.2)	99 (79.2)	0.003

Abbreviations: HDL, high-density lipoprotein; HOMA-IR, homeostasis model assessment insulin resistance.

**Figure 1:**
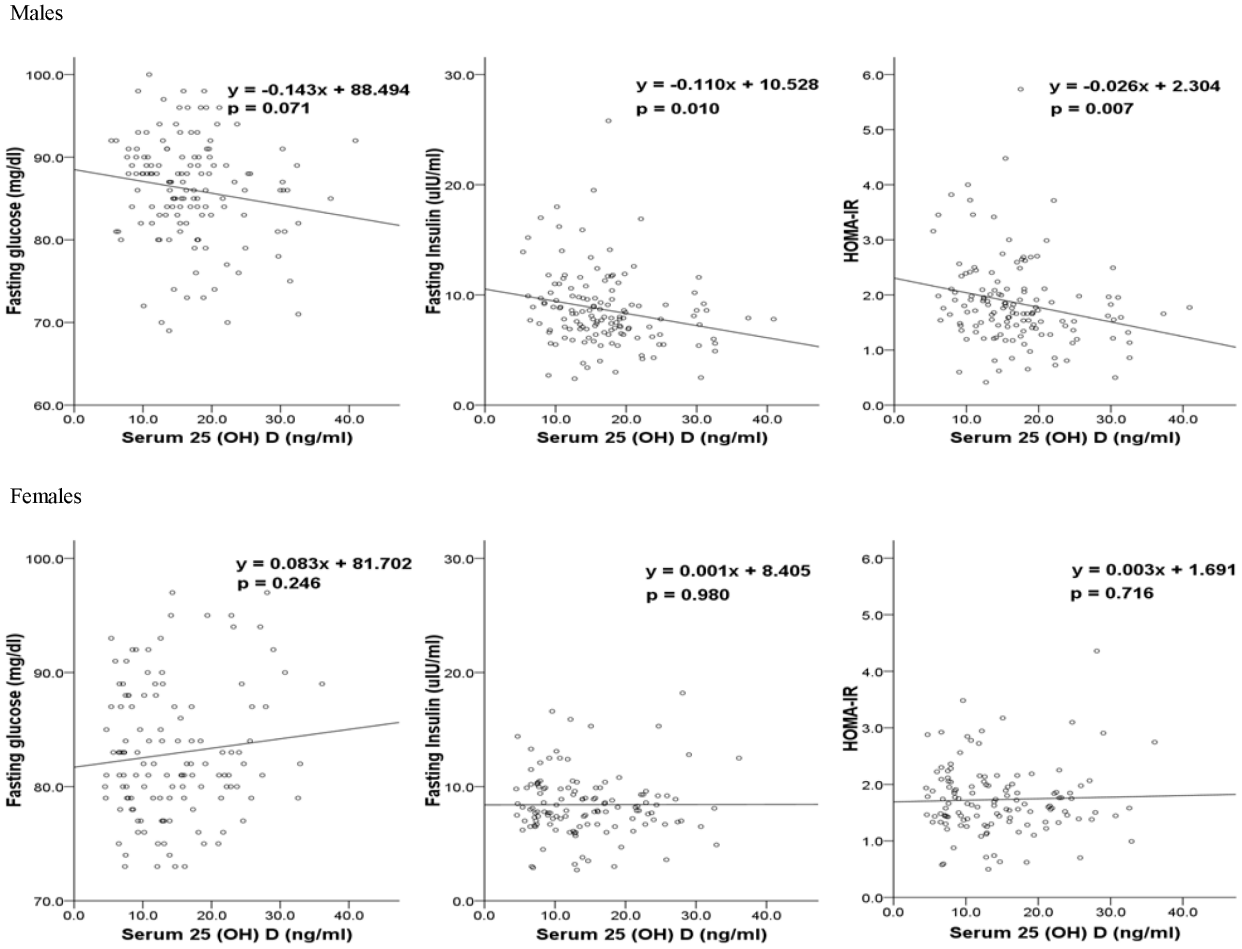
Association between serum 25(OH)D level and glucose metabolism. Upper panels scatter plots with fitted regression lines for 25(OH)D versus fasting glucose, fasting insulin, and HOMA-IR, respectively in males. Lower panels are corresponding scatter plots with fitted regression lines in females. P-values are for the regression coefficients.

Table 2 shows correlations between serum 25(OH)D and other metabolic variables. Serum 25(OH)D level was inversely correlated with total cholesterol (r = −0.213, p = 0.013), fasting insulin (r = −0.256, p = 0.003), and HOMA-IR (r = −0.263, p = 0.002) in males, but not in females. Figure 1 shows clear sex differences in the linear relationship of serum 25(OH)D with fasting glucose, insulin, and insulin resistance. Serum 25(OH)D level was significantly and inversely associated with fasting insulin and HOMA-IR only in male adolescents.

**Table 2:** Correlations between serum 25(OH)D and metabolic characteristics.

	BMI	SBP	DBP	TC	HDLC	Triglyceride	Glucose	Insulin	HOMA-IR	25(OH)D
BMI	-	0.412***	0.062	0.000	−0.218*	0.240**	0.087	0.330***	0.301***	0.028
SBP	0.362***	-	0.468***	0.073	0.010	0.045	0.063	0.143	0.123	0.040
DBP	0.077	0.610***	-	−0.059	0.021	0.020	−0.116	0.120	0.084	0.038
TC	0.156	0.025	−0.060	-	0.353***	0.226**	0.077	0.033	0.049	−0.213*
HDLC	0.046	−0.019	−0.051	0.370***	-	−0.329***	0.065	−0.098	−0.085	0.016
Triglyceride	0.004	0.061	0.039	0.241**	−0.276**	-	0.053	0.147	0.152	−0.148
Glucose	0.100	0.036	0.084	−0.040	0.176*	0.046	-	0.233**	0.410***	−0.153
Insulin	0.302***	0.226*	−0.001	−0.018	−0.102	0.150	0.191*	-	0.975***	−0.256**
HOMA-IR	0.286***	0.210*	−0.002	−0.035	−0.047	0.130	0.400***	0.966***	-	−0.263**
25(OH)D	0.059	−0.024	0.090	−0.022	0.063	−0.101	0.028	−0.049	−0.049	-

Abbreviations: BMI, body mass index; SBP, systolic blood pressure; DBP, diastolic blood pressure; TC, total cholesterol; HDLC, high-density lipoprotein cholesterol; HOMA-IR, homeostasis model assessment insulin resistance. Males, above the diagonal; Females, under the diagonal. * *p*<0.05; ** *p*<0.01; *** *p<*0.001.

Table 3 shows fasting plasma glucose, insulin, and HOMA-IR levels according to serum 25(OH)D. Male adolescents in the lowest 25(OH)D quartile, when compared to those in the highest quartile, had significantly higher fasting glucose (p = 0.039), fasting insulin (p = 0.012), and HOMA-IR (p = 0.008) levels, even after adjustment for age and BMI. There were also increasing trends in fasting glucose (adjusted p for trend  = 0.060), fasting insulin (adjusted p for trend  = 0.009), and HOMA-IR (adjusted p for trend  = 0.007) according to the decrease of 25(OH)D. When the 25(OH)D level was analyzed as a continuous variable, every 10 ng/ml lower level was associated with a 1.44 mg/dl increase in fasting glucose, 1.02 uIU/mL increase in fasting insulin, and 0.25 unit increase in HOMA-IR among male adolescents. However, in female adolescents, serum 25(OH)D level was not associated with fasting glucose, insulin, or insulin resistance.

**Table 3:** Serum 25(OH)D levels and fasting plasma glucose, insulin, and insulin resistance.

Serum 25(OH)D, ng/ml	Glucose, mg/dl			Insulin, uIU/ml			HOMA-IR		
	Means ± SE	Difference*	*p*	Means ± SE	Difference*	*p*	Means ± SE	Difference*	*p*
Males									
4th quartile (19.6 to 40.9, n = 34)	84.53±1.13	Reference		7.67±0.48	Reference		1.62±0.11	Reference	
3rd quartile (15.9 to 19.3, n = 34)	86.26±1.14	1.72	0.265	8.76±0.67	0.71	0.305	1.88±0.15	0.17	0.282
2nd quartile (12.3 to 15.8, n = 33)	85.61±1.13	1.27	0.414	8.40±0.58	1.18	0.090	1.79±0.13	0.27	0.091
1st quartile (5.4 to 12.2, n = 34)	87.71±0.92	3.25	0.039	9.83±0.59	1.77	0.012	2.14±0.14	0.43	0.008
Continuous measure (per 10 ng/ml decrease)		1.44	0.071		1.02	0.004		0.25	0.003
Females									
4th quartile (18.9 to 36.1, n = 32)	84.13±1.11	Reference		8.54±0.51	Reference		1.79±0.12	Reference	
3rd quartile (12.9 to 18.5, n = 33)	81.55±0.99	−2.57	0.075	7.53±0.45	−0.86	0.176	1.52±0.09	−0.24	0.094
2nd quartile (8.4 to 12.8, n = 29)	82.79±1.08	−1.51	0.311	9.15±0.54	0.63	0.338	1.88±0.12	0.09	0.535
1st quartile (4.5 to 8.3, n = 31)	83.16±0.91	−0.74	0.611	8.55±0.46	0.09	0.887	1.76±0.10	−0.01	0.967
Continuous measure (per 10 ng/ml decrease)		−0.86	0.231		0.02	0.953		−0.02	0.752

Abbreviations: HOMA-IR, homeostasis model assessment insulin resistance.

*Adjusted for age and body mass index.

Table 4 presents the association between serum 25(OH)D level and increased insulin resistance, which was defined as a HOMA-IR value higher than the 75^th^ percentile. In male adolescents, the lowest 25(OH)D quartile was significantly associated with increased insulin resistance, and this association was consistent when unadjusted (odds ratio 4.06; 95% confidence interval 1.26 to 13.07), adjusted for age and body mass index (odds ratio 3.59; 95% CI 1.03 to 12.57), and adjusted for age, body mass index, smoking, drinking, and regular exercise (odds ratio 3.54; 95% CI 1.01 to 12.41). Continuous measure of 25(OH)D level was also significantly and inversely associated with insulin resistance among male participants. However, this association was not observed among female participants.

**Table 4:** Serum 25(OH)D level and the risk of increased insulin resistance.

Serum 25(OH)D, ng/ml	No. (%) of increased insulin resistance*	Odds ratio (95% confidence interval) for increased insulin resistance*					
		Unadjusted		Adjusted for age and BMI		Multiple adjusted**	
Males							
4th quartile (19.6 to 40.9, n = 34)	5 (14.7)	1.00		1.00		1.00	
3rd quartile (15.9 to 19.3, n = 34)	9 (26.5)	2.09	(0.62 to 7.05)	1.80	(0.50 to 6.52)	1.87	(0.51 to 6.84)
2nd quartile (12.3 to 15.8, n = 33)	6 (18.2)	1.29	(0.35 to 4.72)	1.45	(0.37 to 5.64)	1.35	(0.34 to 5.34)
1st quartile (5.4 to 12.2, n = 34)	14 (41.2)	4.06	(1.26 to 13.07)	3.59	(1.03 to 12.57)	3.54	(1.01 to 12.41)
Continuous measure (per 10 ng/ml decrease)		2.37	(1.17 to 4.79)	2.29	(1.09 to 4.82)	2.22	(1.04 to 4.74)
Females							
4th quartile (18.9 to 36.1, n = 32)	7 (21.9)	1.00		1.00		1.00	
3rd quartile (12.9 to 18.5, n = 33)	5 (15.2)	0.64	(0.18 to 2.27)	0.62	(0.17 to 2.32)	0.58	(0.15 to 2.19)
2nd quartile (8.4 to 12.8, n = 29)	9 (31.0)	1.61	(0.51 to 5.07)	1.52	(0.46 to 5.01)	1.66	(0.48 to 5.70)
1st quartile (4.5 to 8.3, n = 31)	11 (35.5)	1.96	(0.64 to 6.00)	2.34	(0.73 to 7.50)	1.76	(0.53 to 5.83)
Continuous measure (per 10 ng/ml decrease)		1.19	(0.67 to 2.10)	1.24	(0.69 to 2.25)	1.15	(0.63 to 2.10)

Abbreviations: BMI, body mass index.

* Defined as homeostasis model assessment insulin resistance ≥75 percentile value (males, 2.09; females, 1.99).

**Adjusted for age, body mass index, smoking, drinking, and regular exercise.

## Discussion

We observed a significant inverse association between serum 25(OH)D level and insulin resistance in apparently healthy male adolescents, but not in female adolescents. To our knowledge, this is the first study demonstrating an association between a lower serum 25(OH)D level and increased insulin resistance in healthy Asian adolescents. Moreover, our study population has a narrow age range (15 to 16 years), is relatively lean (mean body mass index 21.7 kg/m^2^), and free of metabolic disorders. These characteristics minimized the possible influences of aging, obesity, and comorbidity in the relationship between vitamin D status and insulin resistance. Most of previous studies on the association between vitamin D and insulin resistance were performed in adult populations [23]–[27]. A few observational studies and clinical trials reported inverse associations between serum 25(OH)D and insulin resistance among overweight or obese adolescents [16]–[19]. On the other hand, no association between serum 25(OH)D and insulin resistance was observed among high school students in Turkey [20]. A recent study reported a sigmoid-shaped association between vitamin D and insulin resistance [27]. The inverse association between 25(OH)D and insulin resistance was observed only at serum 25(OH)D range from 16 to 36 ng/ml, but not at 25(OH)D values higher than 36 ng/ml or lower than 16 ng/ml [27]. However, in our data, we could not observe such a sigmoid-shaped association, probably because of small sample size or narrow age distribution.

Our participants had relatively low serum 25(OH)D levels. Mean serum 25(OH)D was 15.8 ng/ml, while mean serum 25(OH)D level was reported as 24 ng/ml among U.S. adolescents aged 12 to 19 years [28]. Korean high school students devote large amounts of time to studying, and many attend supplemental after-school instruction [29], thus they may not receive sufficient sunlight exposure for adequate cutaneous production of vitamin D. However, we did not collect data on the amount of sunlight exposure. Health effects of insufficient outdoor activity and sunlight exposure need to be further investigated among Korean adolescents. In this study, serum 25(OH)D level was associated with insulin resistance in males, but not in females. This finding suggests that female adolescents, even with vitamin D insufficiency, maintain their insulin sensitivity. The developmental and hormonal differences may explain, at least in part, the sex difference in the associations between serum 25(OH)D level and insulin resistance. Hormonal status during puberty, especially for female adolescents, might affect glucose metabolism. The increase in growth hormone during puberty may contribute to insulin sensitivity via the growth hormone's effect on increasing lipolysis and free fatty acid concentration [30], [31]. In addition, a rise in estrogen level in females may suppress secretion of glucose and protect the pancreatic insulin response to glucose [32]. The relationship between vitamin D status and insulin resistance needs to be further investigated across different sex and age groups.

Several limitations of our study should be acknowledged. First, this is a cross-sectional study, thus the causal inference might be limited. Second, we measured serum 25(OH)D level at a single point, and therefore did not assess seasonal variation. However, therefore seasonal variation is unlikely to distort the relationship between 25(OH)D and insulin resistance, because the health examination was performed over a relatively short period. Third, we could not assess the effects of vitamin D supplements. Our study population consisted of healthy high school students, and none of them reported intake of vitamin D supplements. However, we could not exclude the possible intake of a multivitamin formula including vitamin D. Fourth, objective data on sun exposure and time spent outdoors were not available. Thus, we could not investigate the effects of sunlight on the relationship between vitamin D status and insulin resistance. Finally, the study was conducted at a single high school and included one ethnic group, so the study findings cannot be generalized to other adolescent populations.

In conclusion, this study suggests that lower serum 25(OH)D levels may be associated with increased insulin resistance among healthy male adolescents. Sex differences in the association between serum 25(OH)D and insulin resistance need to be further investigated.
